# Comparative Analysis of Acute-Phase Protein Profiles in Cats Undergoing Ovariectomy: Laparoscopic vs. Conventional Surgery in Short Time After Procedure

**DOI:** 10.3390/ani14223274

**Published:** 2024-11-14

**Authors:** Belén Cuervo, Katy Satué, María Gemma Velasco-Martínez, Joaquín Jesús Sopena, José María Carrillo, Elena Damiá, Deborah Chicharro, Pau Peláez, Emma Martins, Juan Diego García-Martínez, Mónica Rubio

**Affiliations:** 1 Bioregenerative Medicine and Applied Surgery Research Group, Department of Animal Medicine and Surgery, CEU Cardenal Herrera University, CEU Universities, C/Tirant lo Blanc, 7, Alfara del Patriarca, 46115 Valencia, Spain; ksatue@uchceu.es (K.S.); jcarrill@uchceu.es (J.M.C.); elena.damia@uchceu.es (E.D.); debora.chicharro@uchceu.es (D.C.); pau.pelaez@uchceu.es (P.P.); emma.martins1@uchceu.es (E.M.); mrubio@uchceu.es (M.R.); 2 Interdisciplinary Laboratory of Clinical Analysis (INTERLAB-UMU), Department of Animal Medicine & Surgery, Veterinary School, Regional Campus of International Excellence ‘Campus Mare Nostrum’, University of Murcia, Campus de Espinardo, Espinardo, 30100 Murcia, Spain; juandi@um.es

**Keywords:** acute-phase proteins, feline, laparoscopy, ovariectomy, surgical trauma

## Abstract

Acute-phase proteins (APPs) are biomarkers of systemic inflammation, which allow monitoring the response to surgery and post-operative complications. Ovariectomy (OVE) can be a useful model to evaluate surgical trauma and inflammation in feline species. The objective of this study was to evaluate and compare the acute-phase response (APR) after applying two different OVE techniques by measuring serum levels of serum amyloid A (SAA), haptoglobin (Hp), total serum proteins (TSPs), albumin (ALB), and paraoxonase-1 (PON-1) during the first 48 h post-surgery (hospital stay). Forty-two female cats were selected for elective spaying. The animals were randomly distributed into two groups: laparoscopic OVE (L-OVE) and midline OVE (M-OVE). Serum SAA, Hp, TSPs, ALB, and PON-1 were measured before surgery and 1, 6, 12, 24, and 48 h post-surgery. Unlike negative APPs such as PON-1 and ALB, SAA is the most sensitive positive APP for monitoring the inflammatory response in cats undergoing OVE following laparoscopic and conventional surgery. OVE performed in cats via both laparoscopic and midline techniques results in a significative increase in SAA levels, with early recovery, minor variations in serum total protein, and no significant changes in albumin, haptoglobin, or PON-1 over a 48-h period post-procedure. Based on SAA concentrations, the response to surgical trauma does not differ between surgical techniques.

## Simple Summary

Finding new methods to monitor the individual response to surgery and detect post-operative complications is crucial in veterinary practice. Acute phase proteins are biomarkers of systemic inflammation that can be measured for this purpose. Since ovariectomy is a commonly performed surgery and could be a model of surgical trauma and inflammation in cats, the objective of this study was to evaluate and compare the acute-phase response after applying two different techniques: laparoscopic and conventional midline ovariectomy in a short time after the procedure (hospital stay). Forty-two female cats were selected for elective spaying and were randomly distributed into two groups: laparoscopic (L-OVE) and midline ovariectomy (M-OVE). Serum levels of serum amyloid A, haptoglobin, total serum proteins, albumin, and paraoxonase-1 were measured before surgery and 1, 6, 12, 24, and 48 h post-surgery. The results show that serum amyloid A is the most sensitive positive acute-phase protein for monitoring the immediate post-surgical (up to 48 h) inflammatory response in cats undergoing ovariectomy following laparoscopic and conventional surgery. Therefore, the response to surgical trauma does not discriminate between surgical techniques based on serum amyloid A concentrations.

## 1. Introduction

Ovariectomy (OVE) is a widely performed technique in veterinary clinics to control the feline population, prevent the spread of inherited diseases, eliminate undesirable behaviors, and reduce the risk of uterine and ovarian tumors, pyometra, and glandular cystic uterine hyperplasia with secondary infection leading to chronic metritis. Various surgical techniques, including traditional midline, lateral flank, and laparoscopic OVE, are used for these purposes [1,2,3,4]. Surgical stress triggers an acute-phase response (APR). APR begins when cells involved in the innate immune response produce and release pro-inflammatory cytokines, mainly interleukin (IL)-6, IL-1, and tumor necrosis factor α (TNFα) at inflammatory sites. In response to APR, acute-phase proteins (APPs) are synthesized in the liver. These are classified as positive if its serum concentration increases or negative if it decreases in response to inflammation. Positive APPs include serum amyloid A (SAA), alpha-1 acid glycoprotein (AGP), haptoglobin (Hp), and fibrinogen. Negative APPs comprise albumin (ALB), transferrin, apolipoprotein A1, retinol and cortisol binding protein, and paraoxonase-1 (PON-1) [5,6,7]. Based on the magnitude of the response to the inflammatory stimulus, SAA and AGP are considered major APPs, Hp is a moderate APP, and ceruloplasmin (Cp) is a minor APP in cats [8,9,10,11].

Diverse feline studies reported that serum/plasma APPs concentrations increase in a variety of pathologies such as inflammatory and infectious diseases, endocrinological disorders, trauma, hospitalization, tumors, heart diseases, and renal failure in cats [8,11,12,13,14]. Regarding the evaluation of the postoperative inflammatory response based on SAA and Hp, most of the studies have focused on urinary diversion [15], gastrotomy [1], and ovariectomy (OVE) following laparoscopic (L-OVE) and conventional midline procedures (M-OVE) [3,15,16,17]. Although postoperative inflammation in cats subjected to ovariohysterectomy (OHE) following conventional surgery [1,18] has been evaluated, there is no evidence on the monitoring of APR in cats undergoing OVE when comparing two surgical techniques: laparoscopy and conventional surgery. PON-1 circulates in blood bound to high-density lipoproteins (HDL) such as apolipoprotein-1 [19]. During inflammation and oxidative stress, HDL particles lose apolipoprotein-1 and other enzymes, including PON-1, to be replaced by positive AAPs [20,21]. In addition, hepatic synthesis of PON-1 is reduced by inflammation. Some recent studies report a decrease in PON-1 activity in severe infectious processes such as feline infectious peritonitis [22,23]. Although the activity of this enzyme has been analyzed in bitches subjected to OVE [24], its behavior in post-surgical monitoring in cats is unknown.

Therefore, the objective of this study was to evaluate and compare the effects of two surgical techniques: laparoscopic and midline OVE, on the positive APPs SAA and Hp and the negative APPs ALB and PON-1 in a short time after the procedure (during hospital stay). The evaluation of dynamic changes in APPs could help clinicians to choose the least invasive technique and to understand the possible complications that can arise during and after OVE.

## 2. Materials and Methods

### 2.1. Animals

This study was approved by the Animal Experimentation and Ethics Committee (CEEA) of the CEU Cardenal Herrera University (code CEEA 17/004).

The study was conducted with female domestic cats (*Felis silvestris catus*) of various breeds and ages. These cats were presented for elective spaying by their owners from October 2017 to July 2018. All the animals underwent a pre-anesthetic assessment, which included a complete physical examination, thoracic radiographs, and a comprehensive blood analysis. This analysis covered both hematological and biochemical parameters, as well as an ionogram. Cats with significant deterioration in their physical or functional condition were excluded, as were animals with serious alterations in their blood analysis or thoracic radiographs. Only female cats that had elective OVE and were classified as ASA I were included in the study.

### 2.2. Study Groups

Two groups were considered in the study. The animals were randomly included in one of the following groups:

- Group L-OVE: Female cats undergoing laparoscopic OVE with 2 midline working portals (*n* = 21).
- Group M-OVE: Female cats submitted to conventional midline OVE (*n* = 21).

### 2.3. Preparation of the Patient and Anesthetic Protocol

Twelve hours prior to the surgical procedure, all animals were brought to the Veterinary Clinical Hospital of CEU Cardenal Herrera University, with a twelve-hour solid and a two-hour liquid fast. On the same day of surgery, each patient underwent sedation adjusted to the weight of each animal. The sedation protocol consisted of an intramuscular combination of dexmedetomidine 12 μg/kg (Dexdomitor^®^ Zoetis, Madrid, Spain), methadone 0.3 mg/kg (Semfortan^®^ Esteve, Barcelona, Spain), and midazolam 0.3 mg/kg (Midazolam^®^ Normon, Madrid, Spain). The abdominal area of each animal was shaved and disinfected with aseptic cleaning. Additionally, the jugular area was aseptically prepared to place a central venous catheter (Certofix^®^ Braun 22 G, Kronberg, Germany) for blood sample extraction at the study time points.

Once the animal was prepared, anesthesia was induced intravenously with propofol (Propofol Lipuro^®^ Braun) at an effective dose, allowing endotracheal intubation once the swallowing, palpebral, and corneal reflexes were absent. Anesthetic maintenance was performed with sevoflurane (Sevoflo^®^ Abbott, Chicago, IL, USA) in 100% oxygen via an anesthetic machine with a semi-closed circular circuit and corrugated tubes adapted to each patient’s size. Before initiating the surgical procedure, a single prophylactic antibiotic treatment of 20 mg/kg cefazolin (Cefazolin Normon EFG^®^ by Normon) was administered to the patient. Postoperatively, methadone was prescribed as an analgesic at a dose of 0.3 mg/kg every 6 h for 24 h.

### 2.4. Surgical Protocol

All surgeries were performed by the same surgeon with more than 10 years of experience, supported by a veterinarian assistant. Standardized surgical protocols were used, and the duration of each procedure was recorded from the initial skin incision to complete closure. To minimize the impact of the surgical procedure on the results between the groups, in both groups the 5 mm and 37 cm Ligasure bipolar sealing device (LigaSure^TM^ Maryland, Covidien, Dublin, Ireland) was used for vascular sealing and to secure the ovarian supporting tissues. Following surgery, all animals remained hospitalized for 48 h.

- **Ovariectomy by laparoscopy:**

Once the patient was placed in the dorsal decubitus position, the modified Hasson technique was used to establish the pneumoperitoneum [25]. An incision was made 1 cm caudal to the umbilicus, through which the first portal was created. A 6 mm light trocar (Karl Storz, Chiyoda, Tokyo) was then inserted, and once the trocar was placed, the abdomen was insufflated with CO_2_ at a maximum pressure of 5 mmHg. Following this, the second trocar was introduced using the Ternamian visual technique in a medial area between the xiphoid cartilage and the umbilicus using the 3.9 mm light trocar (Karl Storz). After the second portal placement, an abdominal exploration was conducted through the most cranial trocar using the Hopkins panoramic front view optic (14 cm length and 3 mm diameter, Karl Storz), facilitating ovary identification. The animals were then repositioned laterally to identify the most dorsally positioned ovary, which was grasped with Kelly Clickline grasping and dissecting forceps (Karl Storz). It was percutaneously secured in place using a 3/0 glyconate transabdominal suture (Monosyn^®^ Braun), which was extracorporeally fixed with a Halstead forceps to isolate the ovarian pedicle.

Then, the grasping forceps were removed and the Ligasure bipolar sealing device was introduced to provide hemostasis and sectioning of the ovarian supporting tissues. The same procedure was performed with the contralateral ovary, and once the two ovaries were sealed, both were removed through the 6 mm trocar. Finally, the two portal orifices were sutured in layers with the same type of 3/0 monofilament absorbable suture that was used to suspend the ovary.

- **Ovariectomy by conventional laparotomy**

After positioning the patient in the dorsal decubitus position, a 3.5 cm longitudinal incision was made in the cranial abdomen, starting 1 cm caudal to the umbilicus. Once the ovaries were located, the Ligasure bipolar sealing device was used to provide hemostasis and sectioning of the ovarian support tissues. After removing the first ovary, the same procedure was performed on the contralateral ovary. Finally, the muscle layer, the subcutaneous tissue, and the skin were closed in layers with continuous monofilament resorbable glyconate 3/0 monofilament suture (Monosyn^®^ Braun).

### 2.5. Sample Collection

Blood samples were collected from the study animals at the Veterinary Clinical Hospital of CEU Cardenal Herrera University. A total of six 2 mL blood samples were obtained from the central venous catheter and deposited into anticoagulant-free tubes at specified time points: before (PRE-S), immediately after (POST-S), and at 6, 12, and 24 h after completion of surgery.

### 2.6. Sample Analysis

Serum was obtained by centrifuging the blood samples at 3000 rpm for 10 min. It was then stored at −20 °C in Eppendorf tubes. Finally, these tubes were sent to the Interdisciplinary Laboratory of Clinical Analysis (Interlab-UMU) at the University of Murcia for processing.

SAA concentrations were estimated using a previously validated method for cats [26]. The method employed a human turbidimetric immunoassay adapted for an automated analyzer (Olympus AU600, Olympus Mishima, Shizuoka, Japan). The limit of detection was 0.38 μg/mL, and serum concentrations below 5 μg/mL were considered normal for cats [26].

Serum Hp concentrations were measured using the hemoglobin-binding method with a commercial kit (Tridelta Development Ltd., Brey, Ireland). This method has been previously validated in cats [27]. The limit of detection was 0.0088 g/L, and serum concentrations below 3 g/L were considered normal.

TSP concentration was estimated by the biuret method, using commercial reagents (Labtest Diagnóstica, Lagoa Santa, Brazil). Serum ALB was analyzed following the instructions provided by the commercially available kit (Albumin OSR 6102; Olympus Life and Material Science Europe GmbH, Irish branch, Ennis, Ireland).

Regarding PON-1 analysis, serum aryl esterase activity was determined by measuring the hydrolysis of p-nitrophenyl acetate to p-nitrophenol. This measurement was based on the inhibition of enzymatic hydrolysis of 4-nitrophenyl acetate by phenyl acetate [28]. A modification was made to remove the substrate from the reagent buffer and prepare it in water as a separate starting reagent, which remained colorless [29].

P-nitrophenyl acetate undergoes spontaneous hydrolysis in the original system, resulting in a yellowish appearance of the reagent. To initiate the kinetic reaction, the starting reagent was added. Since p-nitrophenyl acetate is not soluble in water, 63 mg of this compound was dissolved in 10 mL of methanol and stored at 2 °C to 8 °C. This solution remains stable for approximately 1 week. Then, 1 mL of the solution was added to 20 mL of distilled water with strong stirring agitation to prevent precipitation. The aqueous solution was prepared each day.

### 2.7. Statistical Analysis

At the end of the study, and once all the data had been collected, statistical analysis was performed using SPSS software for Mac (version 20.0, SPSS Inc., Chicago, IL, USA). A significance level of *p* < 0.05 was considered statistically significant for all cases.

Quantitative variables underwent normality testing using the Shapiro–Wilk test and assessment of homogeneity of variances using the Levene test. Two parameters were evaluated: time (to observe the evolution of the APPs over time) and type of surgery (comparing acute-phase protein results between groups at different study time points). Within each study group, the results obtained at each time point were independently compared to assess the evolution of different APPs.

If variables did not meet normality assumptions, the Kruskal–Wallis non-parametric test was used for comparisons at each time point. For normally distributed variables, an ANOVA test followed by the Tukey post hoc test was performed.

## 3. Results

### 3.1. Animals

Cats enrolled in the study ranged from 9 months to 7 years, while the weights of the animals in groups L-OVE and M-OVE were 2.97 ± 0.76 kg and 2.72 ± 0.46 kg (mean: 2.84 ± 0.63 kg) respectively, without statistical differences between them.

### 3.2. Duration of the Surgery

The duration of the surgery was significantly higher in group L-OVE compared with group M-OVE (*p* < 0.001). The mean surgical time in the L-OVE group was 34.4 ± 11.7 min, while in the M-OVE group it was 21.4 ± 6.49 min.

### 3.3. Acute Phase Proteins

**Figure 1:**
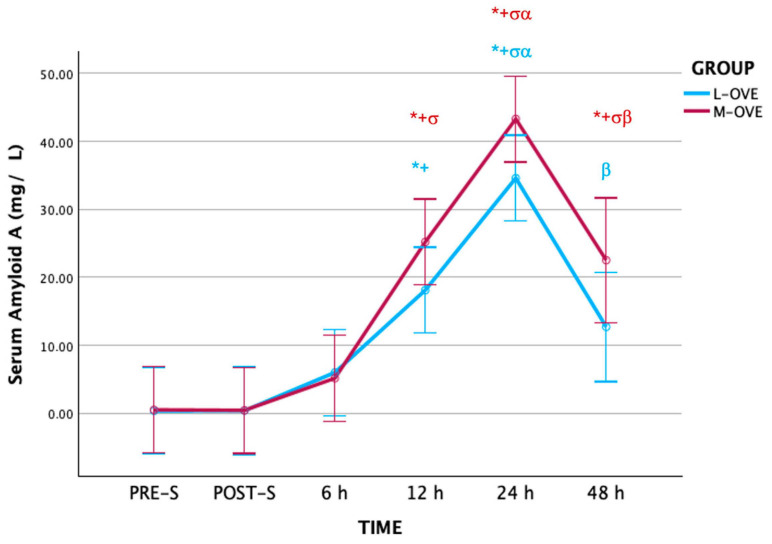
Serum amyloid type A concentration along the experimental period. L-OVE: laparoscopic ovariectomy, M-OVE: midline ovariectomy. The symbols added to the figure indicate significant differences between groups: *: differences with pre-surgical time; +: differences with post-surgical time; σ: differences with 6 h; α: differences with 12 h; β: differences with 24 h.

In both groups, M-OVE and L-OVE, SAA values at 12 h were significantly higher than PRE-S (*p* ≤ 0.001 and *p* ≤ 0.002, respectively) and POST-S (*p* ≤ 0.001 and *p* ≤ 0.002, respectively), and the same as at 24 h compared to PRE-S (*p* ≤ 0.001 and *p* ≤ 0.001, respectively) and POST-S (*p* ≤ 0.001 and *p* ≤ 0.001, respectively). SAA at 48 h in group M-OVE was significantly higher than PRE-S (*p* ≤ 0.002), POST-S (*p* ≤ 0.002), and 6 h (*p* ≤ 0.035), and significantly lower at 48 h compared to 24 h (*p* ≤ 0.004). In this group, SAA also increased at 12 h (*p* ≤ 0.001) and 24 h (*p* ≤ 0.001) compared to 6 h, and at 24 h compared to 12 h (*p* ≤ 0.01). SAA in group L-OVE increased at 24 h compared to 6 h (*p* ≤ 0.001) and 12 h (*p* ≤ 0.005), and at 24 h was significantly higher than those at 48 h (*p* ≤ 0.001) (Figure 1 and Table 1).

**Figure 2:**
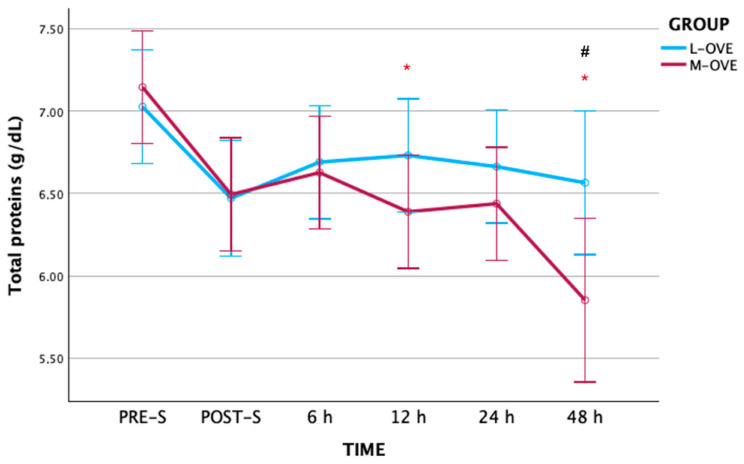
Total serum protein concentrations along the experimental period. L-OVE: laparoscopic ovariectomy, M-OVE: midline ovariectomy. Symbols added to the figure indicate significant differences between groups: *: differences with pre-surgical time; #: differences between groups.

TSPs in group M-OVE were significantly lower at 12 h (*p* ≤ 0.021) and 48 h (*p* ≤ 0.003) compared to PRE-S, and significantly higher in group L-OVE than in group M-OVE at 48 h (*p* ≤ 0.035) (Figure 2 and Table 2).

ALB (Table 3), Hp (Table 4), and PON-1 (Table 5) concentrations did not reveal significant differences between the different sample collection times in both groups (L-OVE and M-OVE). Table 3, Table 4 and Table 5 show the mean values, standard error, and the lower and upper limits of confidence intervals (CI) for serum concentrations of ALB, Hp, and PON-1 at each time and in every group.

## 4. Discussion

To the authors’ knowledge, this study represents the initial concurrent assessment of positive acute-phase proteins (SAA and Hp) and negative acute-phase proteins (ALB, PON-1) in cats undergoing OVE comparing two different surgical approaches, L-OVE and M-OVE, in a short time of 48 h after the procedure (during hospital stay). Given the APR is directly related to the degree of surgical trauma, this study allows an objective assessment of the inflammatory response induced by both methods.

### 4.1. Surgical Time

The mean surgical time in the L-OVE group was significantly higher (34.4 ± 11.7 min) compared to that of the M-OVE group (21.4 ± 6.49 min). A longer surgical time in this species with the L-OVE technique (55.2 ± 16.8 min) compared to midline laparotomy was identified (22.5 ± 5.6 min) [3]. However, the surgical time achieved by L-OVE using electrocoagulation (23 min) [30] was shorter than that obtained in this study. It should be noted that hemostasis induced by electrocoagulation is commonly faster than suture ligation [31]. Since M-OVE has been the commonly used technique in clinical practice, the shorter surgical time in this study may perhaps be associated with the greater experience of the surgeon compared to the L-OVE technique.

### 4.2. SAA

PRE-S SAA concentrations in this study corroborated values previously reported in healthy cats (range: <10 mg/L) [5,17,20,32,33,34]. With regards to the SAA profile, the mean concentrations increased significantly at 6 h, reaching the maximum peak at 24 h, and decreased at 48 h, with values even higher than those of PRE-S and POST-S. These results were similar to those reported in other studies in which OVE was used in this species. In fact, SAA increased between 3 and 6 h [15,17] and at 8 h [18], reaching the peak between 21 and 24 h [15,17], between 24 and 48 h [1], and at 48 h [18]. However, the gradual return of SAA to normal levels took place later in these studies, within 4 days [18] and 5 days [1]. Since the biologic half-life times of SAA are very short (20 to 24 h), once the surgical inflammatory stimulus decreases, SAA returns to basal values within 48 to 72 h [5,6]. Thus, the decrease in SAA levels 24 h after surgery, even taking into account that surgery induces soft tissue inflammation, proves that no early intra- and post-surgical complications arose in these animals, as verified by clinical follow-up until the time of postoperative discharge. As a result, the evaluation of SAA levels could be useful for early detection of inflammatory processes, infections, and massive intra- and postoperative tissue injuries. However, the evolution of this protein beyond 48 h and the existence of long-term complications could not be verified in this study, although no clinical incidents were reported after surgery by the owners.

The highest peak level of SAA concentrations was 40 times greater at PRE-S. This result was higher than that reported by Shida et al. (26.8 times) [1]. In the presence of inflammation, SAA concentrations in blood reaches values between 10 and 100 times higher than basal levels. In addition, this increase is proportional to the intensity of the inflammation induced by the surgery [5,35].

Traumatic injury caused by the surgical procedure results in the release of pro-inflammatory cytokines such as IL-1, IL-6, and TNF α by local monocytes and macrophages. In addition, it stimulates the APR and the synthesis of APPs [36]. In response to the inflammatory stimulus, SAA is synthesized in hepatocytes [5]. Taking into account the fact that it could be possible to have a larger magnitude of the surgical trauma in the cats included in this study, the magnitude of the SAA response in other types of surgeries, such as gastrotomy, was significantly greater than that generated in OHE [1]. Considering that gastrotomy is a more invasive surgical technique than OHE, it seems logical to suggest that the degree of APR and the increase in SAA after gastrotomy is proportional to the severity of the inflammatory damage.

Given the similarity in between the animals included in the study, (age, weight, and good health status) the surgical protocols used in L-OVE and M-OVE and the absence of early perioperative complications, the SAA levels obtained with both surgical techniques suggests that the choice of a specific protocol is not decisive for clinical purposes.

### 4.3. TSPs and ALB

Even though the mean concentrations of TSPs were similar between both protocols in PRE-S, POST-S, 6, 12, and 24 h, it was higher in L-OVE than M-OVE at 48 h. TSPs also decreased significantly at 12 and 48 h in M-OVE compared with PRE-S, with no ALB level modifications. Partially different results of TSP and similar results of ALB levels have been reported in bitches subjected to OVE and OHE. ALB appears to remain stable when these surgical protocols are used, as occurs in bitches. In fact, ALB was not modified either in dogs subjected to OVE using three surgical protocols (laparoscopic, midline, and flank) 1 h after and at 24, 72, and 168 h [24] or in bitches undergoing OHE operations before anesthesia and at 30 min, 60 min, 3, 6, 12, 24 h, and 3 and 7 days post-intervention [37].

In no case did TSPs and ALB exceed the reference ranges for the feline species [35], so any state of hypo- or hyperproteinemia/albuminemia was ruled out. The type and duration of surgical trauma led to greater protein catabolic activity [38]. In fact, ALB is a negative APP, which decreases during APR due to the diversion of amino acids for the synthesis of positive APPs [8]. The stability of ALB and the slight increase in TSPs in the L-OVE group could indicate that the globulin fraction could increase at 48 h. However, given that SAA is currently returning to normal levels, variations in TSPs do not present clinical relevance up to 48 h after surgery.

### 4.4. Hp

Although Hp concentrations did not differ between the procedures or times considered in this study, different results were obtained by other researchers. In fact, Alves et al. showed significantly higher Hp in cats subjected to M-OVE compared to L-OVE, in which the magnitude of the increase was estimated at 117.3% vs. 92.8%, respectively [16]. However, Hp was significantly higher in cats undergoing L-OVE using a miniloop versus Snook hook minilaparotomy [3].

Surgical inflammation is related to trauma, blood loss, and duration of surgery [39]. M-OVE may be harmful if the tissue trauma is extensive, or the surgical incision is long. Alves et al. reported a greater predominance of inflammatory stimuli and contamination of the surgical area in M-OVE compared to L-OVE [16].

However, abdominal CO_2_ insufflation for laparoscopy is associated with visceral pain and peritonitis [40]. Since the duration of surgical time was longer with L-OVE, peritonitis induced by the reaction between CO_2_ and the peritoneal reaction could explain the increase in Hp at 72 h compared to M-OVE, in which a small incision (1.5 cm) and minimal visceral manipulation were performed [3].

In cats, Hp increases 2- to 10-fold in response to inflammation [41]. It is known that APPs are defense molecules that modulate the immune system, transport iron molecules, making them unavailable for bacterial invasion and proliferation, and restore tissue damage caused by the inflammatory stimulus [5,42,43]. The fact that Hp did not increase in either M-OVE or L-OVE could indicate that the degree of tissue trauma has not been intense enough to generate hepatic synthesis of this APP during the 48 h post-surgery period. Additionally, Hp is a moderate APP in this species, so its concentration increases if the trauma generated by surgery is moderate or severe, and will decrease slowly compared to other major proteins such as SAA [20,35].

### 4.5. PON-1

PON-1 concentrations ranged between 2.67 and 4.70 IU/mL without significant fluctuations. These results were lower than those reported for other animals of the same species undergoing surgery [21] and in canines [24], although similar values in bitches [44] have also been reported. Even though this parameter did not vary at 48 h between either method, PON-1 seems to reach higher levels in the M-OVE group compared to the L-OVE group, correlating in both cases, with surgical time.

PON-1 plays a defensive role against oxidative stress (OS) by destroying oxidized lipids, decreasing during inflammation [21]. Oxidized lipids are responsible for the initiation of inflammation and the formation of different cytotoxic and mutagenic substances that can contribute to the appearance of diseases such as feline infectious peritonitis (FIP) [21,23]. Furthermore, PON-1 acts as an endogenous free-radical-scavenging molecule by decreasing the level of systemic OS [21]. Therefore, the fact that this parameter was not subject to modifications could suggest that neither surgical procedure could generate sufficient oxidative stimulus to reduce PON-1, as previously reported in bitches [24], despite the fact that the average values in dogs were higher than those in cats under normal conditions [10].

Based on the use of healthy cats with similar medical histories, following the same surgical protocols and without postoperative complications has made it possible to obtain consistent results. Since M-OVE is a frequently used technique in clinical practice, perhaps it is the greater experience of the surgeons that leads to less associated tissue damage and inflammation and may be the reason for the lack of significant differences between the groups. However, monitoring the response to surgery based on the SAA is relevant for the detection of possible postoperative complications such as inflammation and/or infection.

## 5. Conclusions

OVE in cats following the laparoscopic and midline technique results in an increase and early recovery of SAA, with minor variations in serum total proteins and no changes in albumin, haptoglobin, or PON-1 during a time period of 48 h after the procedure (during hospital stay). Since SAA increases at 12 h and 24 h and decreases at 48 h, SAA analysis can be used as a sensitive tool to monitor the degree of surgical stress and detect intra- or early postsurgical complications (at least up to 48 h) in cats undergoing midline or laparoscopic OVE.

It would be interesting, in future studies, to evaluate these APPs over a longer period of time since early postoperative measurement provides an objective means of predicting the risk of postoperative complications.

## Figures and Tables

**Table 1:** Mean ± SE, confidence interval (CI) and *p*-values of serum amyloid A concentrations in healthy cats during the experimental period in the L-OVE and M-OVE groups.

Serum Amyloid A (mg/L)				Confidence Interval		
Time	Group	Mean	Dev.Error	Lower Limit	Upper Limit	*p*-Value
**Pre-surgical time**	L-OVE	0.352	3.206	−5.966	6.671	0.972
	M-OVE	0.511	3.206	−5.808	6.830	
**Post-surgical time**	L-OVE	0.390	3.285	−6.085	6.865	0.992
	M-OVE	0.438	3.206	−5.881	6.757	
**6 h**	L-OVE	6.000	3.206	−0.319	12.319	0.853
	M-OVE	5.159	3.206	−1.160	11.477	
**12 h**	L-OVE	18.119	3.206	11.800	24.438	0.119
	M-OVE	25.212	3.206	18.894	31.531	
**24 h**	L-OVE	34.595	3.206	28.276	40.914	0.056
	M-OVE	43.303	3.206	36.984	49.622	
**48 h**	L-OVE	12.715	4.075	4.684	20.746	0.114
	M-OVE	22.520	4.646	13.363	31.677	

**Table 2:** Mean ± SE, confidence interval (CI) and *p*-values of total protein concentrations in healthy cats during the experimental period in the L-OVE and M-OVE groups.

Total Proteins (g/dL)				Confidence Interval		
Time	Group	Mean	Dev.Error	Lower Limit	Upper Limit	*p*-Value
**Pre-surgical time**	L-OVE	7.027	0.174	6.683	7.370	0.630
	M-OVE	7.146	0.174	6.802	7.489	
**Post-surgical time**	L-OVE	6.472	0.179	6.120	6.823	0.927
	M-OVE	6.494	0.174	6.151	6.838	
**6 h**	L-OVE	6.691	0.174	6.348	7.034	0.796
	M-OVE	6.627	0.174	6.284	6.971	
**12 h**	L-OVE	6.731	0.174	6.388	7.075	0.167
	M-OVE	6.390	0.174	6.046	6.733	
**24 h**	L-OVE	6.663	0.174	6.320	7.007	0.363
	M-OVE	6.439	0.174	6.095	6.782	
**48 h**	L-OVE	6.565	0.221	6.129	7.002	0.035
	M-OVE	5.853	0.253	5.355	6.351	

**Table 3:** Mean ± SE, confidence interval (CI) and *p*-values of albumin concentrations in healthy cats during the experimental period in the L-OVE and M-OVE groups.

Albumin (g/dL)				Confidence Interval		
Time	Group	Mean	Dev.Error	Lower Limit	Upper Limit	*p*-Value
**Pre-surgical time**	L-OVE	2.795	0.074	2.648	2.941	0.216
	M-OVE	2.925	0.074	2.779	3.072	
**Post-surgical time**	L-OVE	2.570	0.076	2.420	2.721	0.419
	M-OVE	2.657	0.074	2.510	2.803	
**6 h**	L-OVE	2.709	0.074	2.562	2.855	0.613
	M-OVE	2.762	0.074	2.615	2.908	
**12 h**	L-OVE	2.748	0.074	2.601	2.894	0.518
	M-OVE	2.680	0.074	2.533	2.826	
**24 h**	L-OVE	2.666	0.074	2.520	2.813	0.917
	M-OVE	2.677	0.074	2.531	2.824	
**48 h**	L-OVE	2.550	0.095	2.364	2.736	0.873
	M-OVE	2.573	0.108	2.361	2.785	

**Table 4:** Mean ± SE, confidence interval (CI), and *p*-values of haptoglobin concentrations in healthy cats during the experimental period in the L-OVE and M-OVE groups.

Haptoglobin (mg/L)				Confidence Interval		
Time	Group	Mean	Dev.Error	Lower Limit	Upper Limit	*p*-Value
**Pre-surgical time**	L-OVE	4.039	0.271	3.506	4.573	0.680
	M-OVE	3.881	0.271	3.347	4.414	
**Post-surgical time**	L-OVE	3.791	0.277	3.244	4.338	0.680
	M-OVE	3.631	0.271	3.097	4.164	
**6 h**	L-OVE	3.836	0.271	3.303	4.370	0.667
	M-OVE	3.671	0.271	3.138	4.205	
**12 h**	L-OVE	4.004	0.271	3.471	4.538	0.619
	M-OVE	3.814	0.271	3.280	4.347	
**24 h**	L-OVE	4.491	0.271	3.958	5.025	0.739
	M-OVE	4.364	0.271	3.830	4.897	
**48 h**	L-OVE	4.965	0.344	4.287	5.643	0.564
	M-OVE	4.664	0.392	3.891	5.437	

**Table 5:** Mean ± SE, confidence interval (CI), and *p*-values of paraoxonase-1 concentrations in healthy cats during the experimental period in the L-OVE and M-OVE groups.

Paraoxonase 1 (IU/mL)				Confidence Interval		
Time	Group	Mean	Dev.Error	Lower Limit	Upper Limit	*p*-Value
**Pre-surgical time**	L-OVE	3.884	0.202	3.485	4.283	0.145
	M-OVE	4.302	0.202	3.904	4.701	
**Post-surgical time**	L-OVE	3.575	0.207	3.167	3.984	0.207
	M-OVE	3.942	0.202	3.544	4.341	
**6 h**	L-OVE	3.326	0.202	2.927	3.724	0.138
	M-OVE	3.752	0.202	3.353	4.151	
**12 h**	L-OVE	3.325	0.202	2.926	3.724	0.397
	M-OVE	3.568	0.202	3.169	3.966	
**24 h**	L-OVE	3.320	0.202	2.921	3.718	0.292
	M-OVE	3.622	0.202	3.223	4.021	
**48 h**	L-OVE	3.327	0.257	2.820	3.834	0.850
	M-OVE	3.253	0.293	2.675	3.831	

## Data Availability

The datasets used and/or analyzed for this study are available from the corresponding author upon reasonable request.
